# Gestational weight gain, physical activity, sleep problems, substance use, and food intake as proximal risk factors of stress and depressive symptoms during pregnancy

**DOI:** 10.1186/s12884-019-2328-1

**Published:** 2019-05-17

**Authors:** Janina Eichler, Ricarda Schmidt, Andreas Hiemisch, Wieland Kiess, Anja Hilbert

**Affiliations:** 1 Integrated Research and Treatment Center AdiposityDiseases, Medical Psychology and Medical Sociology, Psychosomatic Medicine and Psychotherapy, Leipzig University Medical Center, Philipp-Rosenthal-Strasse 27, 04103 Leipzig, Germany; 2 LIFE Child Leipzig Research Center for Civilization Diseases, University of Leipzig, Philipp-Rosenthal-Strasse 27, 04103 Leipzig, Germany; 3 Department of Women and Child Health, Hospital for Children and Adolescents and Centre for Paediatric Research (CPL), University of Leipzig, Liebigstrasse 20a, 04103 Leipzig, Germany

**Keywords:** Depression, Stress, Health, Risk factors, Pregnancy

## Abstract

**Background:**

Because maternal depressive symptoms and stress during pregnancy are strongly associated with poor health of the mother and the developing child, understanding the predictors of women’s mental health problems is important to prevent complications in the perinatal period. Therefore, this study sought to examine the association between six risk factors – gestational weight gain (GWG), low physical activity, sleep problems, alcohol use, cigarette smoking and snack food intake – and mental health problems during pregnancy. We hypothesized that risk factors would predict mental health problems while adjusting for socio-demographic characteristics and pregnancy intention, both cross-sectionally and longitudinally.

**Methods:**

Hierarchical linear regression analyses were conducted in a population-based sample of *N* = 463 pregnant women during their 2^nd^ trimester (gestational age: 23 to 28 weeks) of whom *n* = 349 were reassessed during their 3^rd^ trimester (gestational age: 33 to 38 weeks). Women had a mean age of 29.8 ± 4.2 years and a mean pregravid body mass index of 23.5 ± 4.3 kg/m^2^. Data were collected by the ‘Leipzig Research Center for Civilization Diseases’ via the Patient Health Questionnaire, Pittsburgh Sleep Quality Index, Food Frequency Questionnaire, self-report items on physical activity and substance use, and objectively measured anthropometrics.

**Results:**

Cross-sectionally, while a higher snack food intake and sleep problems predicted depressive symptoms and stress during the 2^nd^ trimester, gestational weight gain predicted stress only. Longitudinally, sleep problems positively predicted depressive symptoms during the 3^rd^ trimester. All results remained significant after controlling for age, pregravid body mass index, and pregnancy intention. GWG and significant longitudinal effects became insignificant when controlling for gestational age or baseline depressive symptoms and stress, respectively.

**Conclusions:**

The results showed that sleep problems were associated with maternal mental health problems during pregnancy. Longitudinal studies using standardized measures, particularly diagnostic interviews and physiological or biochemical markers, are warranted to confirm patterns of risk factors, their association with depressive symptoms and stress during the course of pregnancy, and their effects on mother’s and child’s health.

**Electronic supplementary material:**

The online version of this article (10.1186/s12884-019-2328-1) contains supplementary material, which is available to authorized users.

## Background

Emotional distress during pregnancy, a time of major transition, was documented in a substantial number of pregnant women [1]. In this regard, depressive disorders, which are particularly characterized by a loss of interest in pleasurable activities and low mood/energy [2], were reported in 9–14% of pregnant women using validated screening instruments or structured interviews, respectively [3]. For more than a decade, research has shown that depressive symptoms during pregnancy were associated with increased maternal and infantile health risks, subsuming complications during pregnancy (e.g., hypertension [4]), adverse pregnancy outcomes (e.g., preterm birth and low birth weight [5]), and long-lasting effects on the child’s and mother’s health (e.g., difficult infant temperament and infant attentional, emotional and behavioral problems [6, 7]). In recent years, a parallel literature on the influence of stress, defined as “a negative emotional experience accompanied by biochemical, physiological and behavioral changes” [8], on the perinatal and long-term maternal and infantile health has quickly grown [9]. Although depressive symptoms may be related to feelings of stress, stress is much more common than depressive symptoms, with 75% of pregnant women self-reporting to feel psychosocially stressed (e.g., financial, work, family or health concerns [10]) during pregnancy. Increasing evidence consistently indicated that stress during pregnancy was related to higher health risks for the mother and the developing child, including perinatal complications (e.g., hypertension, gestational diabetes mellitus, preterm birth, and low birth weight [11–14]) and deficient infantile development over the life course (e.g., respiratory illnesses [15]). To our knowledge, no previous study examined and compared the effects of depressive symptoms and stress on maternal health behaviors during pregnancy in one sample.

Research indicated the link between health-impairing behaviors (e.g., substance use) and both depressive symptoms and stress to be bidirectional [16], although most studies explicitly examined the risk of depressive symptoms and stress on health-impairing behaviors [16]. In fact, there is a lack of evidence on factors contributing to the onset or maintenance of depressive symptoms and stress in pregnant women. In this context, Misra et al. conceptualized the perinatal health framework on distal (e.g., socio-economic status) and proximal (e.g., alcohol use, cigarette smoking, and nutrition) risk factors affecting women’s perinatal health status [17]. Proximal risk factors may be defined as women’s characteristics and behaviors that are variable and modifiable [17]. First and foremost, an individual’s health behaviors can be categorized as proximal, modifiable risk factors for a woman’s health as evidenced in a number of studies in non-pregnant women [18–27]. Specifically in the general adult population, it was found that the objectively measured body mass index (BMI, kg/m^2^), self-reported low physical activity, sleep problems, snack food intake, and cigarette smoking were positively related to depressive symptoms and stress [18–26]. Additionally, alcohol use assessed via self-report questionnaire showed a U-shaped relation with depressive symptoms [27]. However, little is known about these associations in pregnant women [16].

The limited available evidence on the associations between proximal risk factors and depressive symptoms and stress in pregnant women suggested that there was a strong positive relationship between pregravid BMI and levels of depression in women who adequately gained weight during pregnancy according to the 1990 Institute of Medicine recommendations [28] and, to a lesser extent, in pregnant women who excessively gained weight [29]. This suggests that the amount of gestational weight gain (GWG) is associated with depressive symptoms to a lesser extent than weight at conception [29]. In their systematic review including cross-sectional and longitudinal studies, Poudevigne and O’Connor concluded that physical activity during pregnancy was related to better mood which in turn led to better coping with stress [30]. Concerning substance use during pregnancy, alcohol use was found to increase the chance of experiencing depressive symptoms by 1.2 (odds ratio) cross-sectionally [31], while current smokers were at higher risk for later depressive symptoms during pregnancy [32] and experienced elevated stress levels over former and never smokers [33]. Although it may be assumed that nutrition directly or indirectly by weight gain influences mental health [21, 34, 35], to our knowledge, no studies have examined the associations between snack food intake and mental health problems during pregnancy except that loss of control eating was found to be positively associated with depressive symptoms as well as stress during pregnancy [35].

In this context, the aim of this study was to provide prevalence data on depressive symptoms and stress as well as a range of proximal risk factors for mental health problems in pregnant women and to comprehensively elucidate the association between proximal risk factors and mental health problems during pregnancy in a cross-sectional and longitudinal nature. Specifically, we hypothesized that, both cross-sectionally and longitudinally, gestational weight gain, low physical activity, sleep problems, substance use, and snack food intake were significantly positively associated with depressive symptoms and stress. Because distal risk factors such as age, education, occupation, marital status, pregnancy intention, and pregravid BMI were found to be significantly associated with mental health problems in prior studies [29, 36–38], we controlled for these variables while examining hypothesized associations between proximal risk factors and mental health problems.

## Method

### Participants and procedures

This study used data from the ‘Leipzig Research Center for Civilization Diseases (LIFE) Child BIRTH’ study (‘LIFE Child Study’) – a prospective, longitudinal population-based cohort study of children from fetal life until adulthood. The study was conducted according to the Helsinki Declaration [39] and approved by the Ethics Committee of the University of Leipzig, Germany (reg. no. 264-10-19042010). Eligible to participate in the ‘LIFE Child Study’ were all pregnant women in the area of Leipzig (Germany) without further exclusion criteria. Recruitment was carried out via advertisement at different institutions (e.g., hospitals, clinics, public health centers, and doctors’ surgeries) and by media (e.g., internet, radio, and television). At the time of the current study data of 691 pregnant women were available. Information about the study in detail, use of data, potential risks of participation, and the right to withdraw without explanations or adverse consequences at any time from the study was given to all pregnant women. Informed written consent was obtained from all pregnant women. All women underwent comprehensive assessments (e.g., clinical examinations, questionnaires, and interviews) conducted by well-trained assessors (e.g., physicians, nurses, psychologists, and nutritional scientists specifically trained for the study) at visits during the 2^nd^ and 3^rd^ trimester in a research center at the University Hospital of Leipzig. As a compensation for participation, monetary (≤ 20 Euro per child) and some further incentives (e.g., items with the study logo and feedback on results) were given (for detailed information, see [40, 41]). Eligible for the current study were *N* = 463 pregnant women with valid data on the dependent variables from the 2^nd^ trimester assessment of whom *n* = 349 (75.4%) were reassessed at the 3^rd^ trimester visit.

### Dependent variables assessed at the 2^nd^ and 3^rd^ trimester visits

#### Mental health problems

Mental health problems were assessed by corresponding modules of the German version of the Patient Health Questionnaire [42]. The Patient Health Questionnaire [43] is a self-administered questionnaire to screen for stress-related symptoms and mental disorders according to DSM-IV [2]. The depressive symptoms module (PHQ-9) targets the frequency of being bothered by different problems (e.g., depressive mood or hopelessness) during the last two weeks and consists of 9 items (0 = *not at all* to 3 = *nearly every day*). In this sample, the PHQ-9 showed acceptable internal consistency (Cronbach’s α: 2^nd^ trimester = .74, 3^rd^ trimester = .76). The PHQ-9 has shown good convergent validity, high specificity (84%), and sensitivity (85%) in previous research in an US-American sample of 745 pregnant women [44]. The 10-item stress module refers to the frequency of being bothered by different problems (e.g., health worries) during the last four weeks (0 = *not impaired* to 2 = *strongly impaired*). The items of each scale were summed to build separate total scores [42]. Scores of ≥10 were used to detect pregnant women with symptoms of a major depressive disorder [45] or moderate to high psychosocial stress scores [43], respectively.

### Independent variables assessed at the 2^nd^ trimester visit

All independent variables underwent standardization, validation, computerization, and piloting [40, 41].

#### Gestational weight gain

Gestational weight gain (kg) was calculated by subtracting the retrospectively self-reported weight at the beginning of pregnancy from the weight objectively measured with the calibrated scale ‘Seca 701’ (Seca Gmbh & Co. KG, Germany) during the intake sessions. Assuming an average weight gain of 1.6 kg during the 1^st^ trimester [46], weekly GWG during the 2^nd^ trimester was compared to the 2009 Institute of Medicine recommendations for weight gain during pregnancy [47].

#### Physical activity

Physical activity was assessed by one self-report questionnaire item: “*Please state how often you are physically active”* (0 = *less than once a month*, 1 = *once a month*, 2 = *once a week*, and 3 = *daily*).

#### Sleep problems

Sleep problems were assessed by the Pittsburgh Sleep Quality Index (PSQI; [48]), a self-report questionnaire addressing sleep patterns during the last month. Of the 24 items, 18 are used to quantitatively assess seven components of sleep (0 = *no difficulty* to 3 = *strong difficulty*). A global score was built without imputing data if at least six of the seven components of sleep were filled in completely [48]. A cut-off of 5 was used to distinguish good (< 5) from poor sleepers (≥ 5); [48]). In this sample, the PSQI showed an internal consistency of Cronbach’s α = .65. The PSQI has shown good construct validity and acceptable reliability in a previous study of 1488 US-American pregnant women [49].

#### Substance use

Women were asked if they smoked or drank alcohol during pregnancy via interview. Women who denied the intake of these substances (0 = *no, none of this*) were defined as *non-smoker* or *non-drinker*, while those who stopped using cigarettes or alcohol after finding out about the pregnancy (1 = *cigarette smoking* or *alcohol use until pregnancy was known*) were categorized as *quitter*_*smoking*_ or *quitter*_*drinking*_, respectively. Women who currently used cigarettes or alcohol (2 = *current cigarette smoking* or *alcohol use*) were classified as *smoker* or *drinker*, respectively.

#### Snack food intake

Snack food intake was assessed using a mean score of all 8 snack food items concerning the intake of chocolate, chocolate bars, gummy bears, ice cream, cookies, crisps, nuts, and kernels selected from a 163-item self-report Food Frequency Questionnaire [50]. The Food Frequency Questionnaire combines the frequency (0 = *never* to 8 = *at least four times a day*) and the average amount of used portions (1 = ^*1*^*/*_*4*_ *portion* to 5 = *3 portions*) of consumed foods over the preceding four weeks. It has not been validated in pregnant women in Germany yet [50], but comparable Food Frequency Questionnaires have shown good convergent validity (e.g., in a sample of 113 Finnish pregnant women [51]).

### Socio-demographic characteristics

Information on age, marital status, occupation, education, income, and pregravid BMI were obtained through self-report questionnaires during the 2^nd^ trimester visit. Further, the Winkler Index [52] was used to depict the socio-economic status (SES) of the family based on income, education, and occupation, ranging between 3 and 21 (3–8 = low, 9–14 = middle, and 15–21 = high socio-economic status). Additionally, pregnancy intention was assessed via interview by asking the women if the current pregnancy was planned or not planned (0 = *not planned* or 1 = *planned*).

### Statistical analysis

Preparing data, outlier analyses based on Shapiro-Wilks tests of normal distribution were conducted for all variables. Using Chebyshev’s inequality, normally or non-normally distributed data which were 3 or 4, respectively, standard deviations under or above the mean of each variable were eliminated [53]. First, prevalence data and mean scores of depressive symptoms, stress, and proximal risk factors were reported as %, *N*, or *M* (*SD*) for categorical and numerical variables, respectively. Paired *t*-tests were used to compare mean scores of depressive symptoms and stress during the 2^nd^ versus 3^rd^ trimester. Second, to identify covariates for the following analyses, Spearman rank order, Pearson product-moment correlations, and Phi correlation coefficients were calculated between proximal risk factors and mental health problems, as well as socio-demographic characteristics and pregnancy intention, respectively. Because age, pregravid BMI, and pregnancy intention were significantly associated with proximal risk factors (*r* = .121–.257, all *p*s < .05) and mental health problems (*r* = .121–.147, all *p*s < .05), they were included as covariates in all analyses. SES was selectively associated with certain proximal risk factors and mental health problems (*r* = .163–.200, all *p*s < .05) but only available in a subsample of pregnant women (*n* = 306 of 463). In order to avoid data loss, SES was entered as a covariate in supplementary analyses. Marital status was not significantly associated with proximal risk factors and mental health problems. Third, to predict depressive symptoms and stress during the 2^nd^ and 3^rd^ trimester by proximal risk factors assessed during the 2^nd^ trimester, hierarchical multiple linear regression analyses were used. Univariate normality of residuals was tested – no violations were detected. In all regression analyses using cross-sectional data of the 2^nd^ trimester, covariates were entered in the 1^st^ block (age, pregravid BMI, and pregnancy intention) and proximal risk factors were entered in the 2^nd^ block. Concerning the regression analyses including GWG as an independent variable, gestational age assessed during the 2^nd^ trimester (*n* = 330 of 463) was entered in a 3^rd^ block, in order to control for actual gestational week. For longitudinal analyses, covariates were entered in the 1^st^ block (pregravid BMI and pregnancy intention), proximal risk factors were entered in the 2^nd^ block, and mental health problems assessed during the 2^nd^ trimester were entered in the 3^rd^ block, in order to control for baseline mental health problems. Because of individual profiles of missing data on each risk factor, separate hierarchical linear regression analyses were carried out in order to avoid data loss due to reduced sample size. Missing data were not imputed because of the single-item assessment of independent variables. The results were unlikely affected by missing data because they were proportionally distributed (Little’s MCAR-test: χ^2^ (89, *N* = 463) = 90.50, *p* = .436). Therefore, missing data were excluded listwise. *R*^2^ was used to assess the proportion of explained variance in mental health problems through maternal proximal risk factors.

Statistical significance was set to a two-tailed *p* < .05. Statistical analyses were performed using SPSS for Windows (Version 24.0; SPSS, Inc., Chicago, IL). Using the software package G*Power (Version 3.1.9.2; [54]), a post-hoc power analysis showed that the statistical power (1 – β) for the multiple regression analysis during the 2^nd^ trimester (*N* = 463, α = .05, four to seven predictor variables) and the 3^rd^ trimester (*n* = 349, α = .05, three to six predictor variables) was 0.99 to 1.00 for detecting a medium effect (Cohen’s f^2^ = 0.15).

## Results

### Sample characteristics

First, pregnant women who were reassessed at the 3^rd^ trimester visit (*n* = 349) were compared with those who were assessed at the 2^nd^ trimester visit only (*n* = 114). They differed only in GWG with women who were not reassessed having a lower GWG (*p* = .023). No other statistically significant differences between assessment completers and drop-outs were observed (all *p*s > .05). Therefore, socio-demographic characteristics were reported for women assessed at the 2^nd^ trimester visit only. Women were 18 to 41 years old (*M* = 29.8, *SD* = 4.2) and had a mean pregravid BMI of 23.5 ± 4.3 kg/m^2^ ranging from 17.2 to 40.3 kg/m^2^ based on objectively measured height and self-reported pregravid weight (see Table 1). Categorically, 4.8% had underweight (*n* = 22; BMI < 18.5 kg/m^2^), most had normal weight (69.9%, *n* = 323; BMI = 18.5–24.9 kg/m^2^), 17.3% had overweight (*n* = 80; BMI = 25.0–29.9 kg/m^2^), and 8.2% had obesity (*n* = 38; BMI ≥ 30.0 kg/m^2^; [55]). Gestational age ranged between 23 to 28 (*M* = 24.9, *SD* = 1.0, *n* = 334) and 33 to 38 (*M* = 36.0, *SD* = 0.6, *n* = 253) weeks during the 2^nd^ and 3^rd^ trimester, respectively. The majority were primiparas (97.0%, *n* = 449 of 461) and planned their pregnancy (81.2%, *n* = 376). Concerning socio-demographic characteristics, the majority identified themselves as partnered (94.4%, *n* = 320 of 339), employees (71.8%, *n* = 242 of 337), had at least 12 years of education (72.9%, *n* = 247 of 339), and had a monthly income between 2.500 and 4.000 Euro (41.7%, *n* = 110 of 264). Based on the Winkler Index [52], most of the families were categorized as having a middle (47.7%; *n* = 146 of 306) to high (44.8%, *n* = 137 of 306) SES.

**Table 1:** Prevalence rates and mean scores of study variables

	Cross-sectional			Longitudinal					
	2^nd^ trimester (*N* = 463)			2^nd^ trimester (*n* = 349)			3^rd^ trimester (*n* = 349)		
*Variable*	*n*	*M* or *n*	*SD* or %	*n*	*M* or *n*	*SD* or %	*n*	*M* or *n*	*SD* or %
Mental health problems									
Depressive symptoms (PHQ-9)	463	5.09	3.23	349	5.02	3.12	349	4.96	3.08
Stress (PHQ-D)	463	4.00	3.04	349	3.86	2.93	349	3.27	2.81
Proximal risk factors									
Gestational weight gain (kg)	458	7.19	3.82	344	6.88	3.61	–	–	–
Physical activity	342			275			–	–	–
*less than once a month*		91	26.6		76	27.6			
*once a month*		48	14.0		36	13.1			
*once a week*		166	48.5		136	49.5	–	–	–
*daily*		37	10.8		27	9.8			
Sleep problems (PSQI)	292	5.50	3.99	225	5.35	3.69	–	–	–
Alcohol use	399			309			–	–	–
*non-drinker*		293	73.4		221	71.5			
*quitter*		106	26.6		88	28.5			
*drinker*		0	0.0		0	0.0			
Cigarette smoking	364			275			–	–	–
*non-smoker*		296	81.3		224	81.5			
*quitter*		67	18.4		50	18.2			
*smoker*		1	0.3		1	0.4			
Snack food intake (portion)	389	1.33	1.12	292	1.27	0.97	–	–	–
Covariates									
Age (years)	463	29.77	4.18	349	29.75	4.10	–	–	–
Pregravid BMI (kg/m^2^)	463	23.50	4.29	349	23.38	4.31	–	–	–
Pregnancy intention	463			349			–	–	–
*Pregnancy not planned*		87	18.8		61	17.5			
*Pregnancy planned*		376	81.2		288	82.5			

*Note. n* number of pregnant women included in the model. PSQI Pittsburgh Sleep Quality Index. PHQ-9 Patient Health Questionnaire - Depression. PHQ-D Patient Health Questionnaire - German version. BMI body mass index

### Prevalence rates and mean scores of proximal risk factors and mental health problems

There was considerable variability in the prevalence of maternal proximal risk factors during the 2^nd^ trimester (see Table 1). Concerning GWG, 22.3% (*n* = 71) adequately gained weight, 44.5% (*n* = 142) gained too much weight, and 33.2% (*n* = 106) gained too little weight. Furthermore, 40.6% (*n* = 106) reported to be physically active less than once a week. Sleep problems were the risk factor mostly reported (67.1%, *n* = 196). Self-reported cigarette smoking (18.7%, *n* = 68) and alcohol use (26.6%, *n* = 106) at any time during pregnancy were present in approximately a fifth to a quarter of pregnant women, respectively. More than half of the sample reported a daily snack food intake of ≥1 portion (53.2%, *n* = 207).

While the mean stress scores were significantly different between the 2^nd^ and 3^rd^ trimester assessment (*p* < .001), women’s depression scores were not (*p* = .563). During the 2^nd^ and 3^rd^ trimester 11.9% (*n* = 55) and 7.2% (*n* = 30) exceeded the cut-off for a major depressive disorder, respectively. Experiencing at least one stressor was reported by 95.0% (*n* = 440) and 82.2% *(n* = 287) in their 2^nd^ and 3^rd^ trimester, respectively. Moderate to high stress scores during the 2^nd^ and 3^rd^ trimester were prevalent in 6.9% (*n* = 32) and 3.7% (*n* = 13), respectively.

### Prediction of depressive symptoms

The results of the hierarchical multiple linear regression analyses calculated to predict depressive symptoms during the 2^nd^ and 3^rd^ trimester are displayed in Table 2.

**Table 2:** Results of hierarchical multiple linear regression analysis to predict depressive symptoms during the 2^nd^ and 3^rd^ trimester by proximal risk factors of the 2^nd^ trimester

*Variable*	*2* ^*nd*^ *trimester – Cross-sectional*						*3* ^*rd*^ *trimester – Longitudinal*					
	*n*	*B (SE)*	β	*R* ^2^	*F*	*R* ^2^_change_	*n*	*B (SE)*	β	*R* ^2^	*F*	*R* ^2^_change_
GWG	458						344					
Block 1				0.03	4.67**	0.03				0.01	2.24	0.01
Age		−0.06 (0.04)	−0.08					–	–			
Pregravid BMI		0.08 (0.03)	0.11*					0.08 (0.04)	0.10			
Pregnancy intention		−0.79 (0.38)	−0.09*					−0.38 (0.46)	−0.04			
Block 2				0.04	4.22	0.01				0.02	2.02	0.01
GWG		0.07 (0.04)	0.08					0.06 (0.05)	0.07			
Physical activity	342						275					
Block 1				0.02	1.79	0.02				0.01	0.65	0.01
Age		−0.06 (0.04)	−0.08					–	–			
Pregravid BMI		0.05 (0.04)	0.07					0.05 (0.05)	0.07			
Pregnancy intention		−0.44 (0.46)	− 0.05					−0.02 (0.52)	−0.01			
Block 2				0.03	1.79	0.02				0.04	2.04	0.03
Physical activity	reference group: *once a week*											
*Less than once a month*		1.05 (0.42)	0.15					1.23 (0.45)	0.17^**^			
* Once a month*		0.34 (0.52)	0.04^*^					0.30 (0.59)	0.03			
* Daily*		0.14 (0.58)	0.01					0.36 (0.66)	−0.03			
Sleep problems	292						225					
Block 1				0.07	7.02***	0.07				0.02	2.48	0.02
Age		−0.06 (0.05)	−0.08					–	–			
Pregravid BMI		0.12 (0.04)	0.15**					0.08 (0.05)	0.11			
Pregnancy intention		−1.58 (0.49)	−0.18**					− 0.81 (0.56)	− 0.10			
Block 2				0.26	25.32^***^	0.19				0.10	8.55^***^	0.08
Sleep problems		0.38 (0.04)	0.45^***^					0.26 (0.06)	0.30^***^			
Alcohol use	399						309					
Block 1				0.02	4.45**	0.02				0.02	3.69*	0.02
Age		−0.07 (0.04)	−0.09					–	–			
Pregravid BMI		0.10 (0.04)	0.14**					0.11 (0.04)	0.15**			
Pregnancy intention		−0.58 (0.43)	− 0.07					−0.32 (0.50)	−0.04			
Block 2				0.04	3.91**	0.01				0.03	2.81^*^	0.00
Alcohol use	reference group: *non-drinker*											
* Quitter*		−0.56 (0.37)	−0.08					−0.43 (0.42)	−0.06			
Cigarette smoking	364						275					
Block 1				0.02	2.80*	0.02				0.01	1.02	0.01
Age		−0.03 (0.04)	−0.04					–	–			
Pregravid BMI		0.09 (0.04)	0.11*					0.06 (0.05)	0.08			
Pregnancy intention		−0.75 (0.47)	− 0.08					−0.28 (0.57)	−0.03			
Block 2				0.03	2.43*	0.01				0.01	0.95	0.00
Cigarette smoking	reference group: *non-smoker*											
* Quitter*		0.52 (0.45)	0.06					0.48 (0.53)	0.06			
Snack food intake	389						292					
Block 1				0.03	4.23**	0.03				0.03	4.13*	0.03
Age		−0.05 (0.04)	−0.06					–	–			
Pregravid BMI		0.11 (0.04)	0.14**					0.12 (0.04)	0.16**			
Pregnancy intention		−0.54 (0.41)	− 0.07					− 0.42 (0.50)	− 0.05			
Block 2				0.04	4.53^*^	0.01				0.03	3.15^*^	0.00
Snack food intake		0.32 (0.14)	0.12^*^					0.21 (0.19)	0.06			

*Note. n* number of pregnant women included in the model. *B (SE)* estimated value of raw (unstandardized) regression coefficient (standard error). β population value (standardized) of regression coefficient. *R*^*2*^ coefficient of multiple determination. *F* Fisher’s ratio. BMI body mass index, kg/m^2^. GWG gestational weight gain. ^***^p < .001. ^**^p < .01. ^*^p < .05

#### Gestational weight gain

Cross-sectional. Pregravid BMI (*p* = .017) positively and pregnancy intention (*p* = .040) negatively predicted depressive symptoms accounting for 3% of variance. GWG did not predict depressive symptoms during the 2^nd^ trimester significantly.

Longitudinal. Neither the covariates nor GWG of the 2^nd^ trimester predicted depressive symptoms during the 3^rd^ trimester significantly.

#### Physical activity

Cross-sectional. Neither the covariates nor physical activity emerged as significant predictors of depressive symptoms during the 2^nd^ trimester.

Longitudinal. In the prediction of depressive symptoms during the 3^rd^ trimester by physical activity, neither covariates nor physical activity of the 2^nd^ trimester proved to be significant predictors.

#### Sleep problems

Cross-sectional. Pregravid BMI (*p* = .009) positively and pregnancy intention (*p* = .001) negatively predicted depressive symptoms during the 2^nd^ trimester accounting for 7% of variance. Sleep problems (*p* < .001) positively predicted depressive symptoms during the 2^nd^ trimester accounting for 19% of additional variance.

Longitudinal. The covariates were not found to be significant predictors. Sleep problems (*p* < .001) of the 2^nd^ trimester positively predicted depressive symptoms during the 3^rd^ trimester, additionally accounting for 8% of variance. However, after adjusting for depressive symptoms (β = 0.66, *p* < .001) and stress (β = 0.10, *p* = .085) during the 2^nd^ trimester in the 3^rd^ block, which accounted for additional 42% of variance, *F* (5, 224) = 47.84, sleep problems did not predict depressive symptoms during the 3^rd^ trimester anymore (*p* = .957).

#### Alcohol use

Cross-sectional. Pregravid BMI (*p* = .006) significantly positively predicted depressive symptoms accounting for 2% of variance. Alcohol use did not significantly predict depressive symptoms during the 2^nd^ trimester.

Longitudinal. While pregravid BMI (*p* = .009) emerged as a significant positive predictor accounting for 2% of variance, alcohol use of the 2^nd^ trimester did not emerge as a significant predictor of depressive symptoms during the 3^rd^ trimester.

#### Cigarette smoking

Cross-sectional. The only significant predictor was pregravid BMI (*p* = .030) accounting for 2% of variance. Cigarette smoking did not significantly predict depressive symptoms during the 2^nd^ trimester.

Longitudinal. Both the covariates and cigarette smoking of the 2^nd^ trimester did not appear as significant predictors of depressive symptoms during the 3^rd^ trimester.

#### Snack food intake

Cross-sectional. Pregravid BMI (*p* = .005) emerged as a significant positive predictor accounting for 3% of variance. Snack food intake (*p* = .022) positively predicted depressive symptoms during the 2^nd^ trimester, additionally accounting for 1% of variance.

Longitudinal. While pregravid BMI (*p* = .008) significantly positively predicted depressive symptoms during the 3^rd^ trimester, snack food intake of the 2^nd^ trimester did not.

### Prediction of stress

The results of the hierarchical multiple linear regression analyses examined to predict stress during the 2^nd^ and 3^rd^ trimester are shown in Table 3.

**Table 3:** Results of hierarchical multiple linear regression analysis to predict stress during the 2^nd^ and 3^rd^ trimester by proximal risk factors of the 2^nd^ trimester

	*2* ^*nd*^ *trimester – Cross-sectional*						*3* ^*rd*^ *trimester – Longitudinal*					
*Variable*	*n*	*B (SE)*	β	*R* ^2^	*F*	*R* ^2^_change_	*n*	*B (SE)*	β	*R* ^2^	*F*	*R* ^2^_change_
GWG	458						344					
Block 1				0.02	3.06*	0.02				0.03	5.26**	0.03
Age		−0.03 (0.03)	−0.04					–	–			
Pregravid BMI		0.07 (0.03)	0.10*					0.06 (0.03)	0.10			
Pregnancy intention		−0.67 (0.36)	−0.09					−1.01 (0.39)	− 0.14**			
Block 2				0.03	3.99^**^	0.01				0.03	3.54^*^	0.00
GWG		0.10 (0.04)	0.12^*^					0.01 (0.04)	0.02			
Physical activity	342						275					
Block 1				0.00	0.51	0.00				0.01	0.98	0.01
Age		−0.01 (0.04)	−0.01					–	–			
Pregravid BMI		0.04 (0.04)	0.05					0.03 (0.04)	0.04			
Pregnancy intention		−0.28 (0.43)	− 0.04					−0.55 (0.45)	−0.07			
Block 2				0.03	1.86	0.03				0.01	0.84	0.01
Physical activity	reference group: *once a week*											
* Less than once a month*		1.01 (0.39)	0.15^**^					0.43 (0.40)	0.07			
* Once a month*		0.23 (0.48)	0.03					0.65 (0.51)	0.08			
* Daily*		1.16 (0.54)	0.12^*^					0.33 (0.58)	0.04			
Sleep problems	292						225					
Block 1				0.03	2.51	0.03				0.07	7.87***	0.07
Age		−0.01 (0.04)	−0.02					–	–			
Pregravid BMI		0.07 (0.04)	0.09					0.09 (0.04)	0.14*			
Pregnancy intention		−0.97 (0.46)	−0.12*					−1.51 (0.47)	−0.21**			
Block 2				0.09	7.26^***^	0.07				0.09	7.37^***^	0.02
Sleep problems		0.20 (0.04)	0.27^***^					0.12 (0.05)	0.16^*^			
Alcohol use	399						309					
Block 1				0.03	3.85*	0.03				0.04	6.80**	0.04
Age		−0.03 (0.04)	−0.04					–	–			
Pregravid BMI		0.09 (0.03)	0.13*					0.10 (0.04)	0.15**			
Pregnancy intention		−0.74 (0.40)	−0.09					−1.03 (0.42)	−0.14*			
Block 2				0.03	3.05^*^	0.00				0.04	4.69^**^	0.00
Alcohol use	reference group: *non-drinker*											
* Quitter*		−0.28 (0.35)	−0.04					−0.25 (0.35)	−0.04			
Cigarette smoking	364						275					
Block 1				0.02	2.05	0.02				0.02	2.36	0.02
Age		0.02 (0.04)	0.03					–	–			
Pregravid BMI		0.07 (0.04)	0.10					0.07 (0.04)	0.11			
Pregnancy intention		−0.61 (0.44)	−0.07					−0.58 (0.47)	−0.07			
Block 2				0.02	2.16	0.01				0.02	1.59	0.00
Cigarette smoking	reference group: *non-smoker*											
* Quitter*		0.67 (0.42)	0.08					0.12 (0.44)	0.02			
Snack food intake	389						292					
Block 1				0.02	2.02	0.02				0.03	4.23*	0.03
Age		−0.02 (0.04)	−0.03					–	–			
Pregravid BMI		0.07 (0.04)	0.10*					0.07 (0.04)	0.10			
Pregnancy intention		−0.39 (0.39)	−0.05					−0.94 (0.43)	−0.13*			
Block 2				0.04	3.99^**^	0.02				0.03	3.27^*^	0.00
Snack food intake		0.42 (0.13)	0.16^**^					0.19 (0.16)	0.07			

*Note. n* number of pregnant women included in the model. *B (SE)* estimated value of raw (unstandardized) regression coefficient (standard error). β population value (standardized) of regression coefficient. *R*^*2*^ coefficient of multiple determination. *F* Fisher’s ratio. BMI body mass index, kg/m^2^. GWG gestational weight gain. ^***^p < .001. ^**^p < .01. ^*^p < .05

#### Gestational weight gain

Cross-sectional. Pregravid BMI (*p* = .035) positively predicted stress accounting for 2% of variance. GWG (*p* = .010) positively predicted stress, additionally accounting for 1% of variance. Adjusting for gestational age (β = 0.04, *p* = .275) in the subsample with valid data on actual gestational week assessed during the 2^nd^ trimester in the 3^rd^ block, accounting for additional 0.4% of variance, *F* (4, 325) = 3.02, turned the significant predictor GWG insignificant (*p* = .418).

Longitudinal. Pregnancy intention (*p* = .010) negatively predicted stress during the 3^rd^ trimester accounting for 3% of variance. GWG of the 2^nd^ trimester did not emerge as a significant predictor of stress during the 3^rd^ trimester.

#### Physical activity

Cross-sectional. In predicting stress during the 2^nd^ trimester by physical activity, neither covariates nor physical activity proved to be significant predictors.

Longitudinal. Neither the covariates nor physical activity of the 2^nd^ trimester emerged as significant predictors of stress during the 3^rd^ trimester.

#### Sleep problems

Cross-sectional. Pregnancy intention (*p* = .036) negatively predicted stress during the 2^nd^ trimester accounting for 3% of variance. Sleep problems (*p* < .001) positively predicted stress during the 2^nd^ trimester, additionally accounting for 7% of variance.

Longitudinal. Pregravid BMI (*p* = .032) positively and pregnancy intention (*p* = .002) negatively predicted stress accounting for 7% of variance. Sleep problems of the 2^nd^ trimester positively predicted stress during the 3^rd^ trimester (*p* = .015), additionally accounting for 2% of variance. Adjusting for depressive symptoms (β = 0.13, *p* = .043) and stress (β = 0.58, *p* < .001) during the 2^nd^ trimester in a 3^rd^ block, accounting for additional 39% of variance, *F* (5, 224) = 40.11, turned the significant predictor sleep problems insignificant (*p* = .544), but pregnancy intention remained a significant predictor (*p* = .038) accounting for 7% of the variance.

#### Alcohol use

Cross-sectional. Pregravid BMI (*p* = .010) positively predicted stress accounting for 3% of variance. Alcohol use did not emerge as a significant predictor of stress during the 2^nd^ trimester.

Longitudinal. Pregravid BMI (*p* = .008) positively and pregnancy intention (*p* = .015) negatively predicted stress during the 3^rd^ trimester accounting for 4% of variance. Alcohol use of the 2^nd^ trimester did not significantly predict stress during the 3^rd^ trimester.

#### Cigarette smoking

Cross-sectional. In the prediction of stress by cigarette smoking, neither covariates nor cigarette smoking were significant predictors.

Longitudinal. The covariates and cigarette smoking of the 2^nd^ trimester did not emerge as significant predictors of stress during the 3^rd^ trimester.

#### Snack food intake

Cross-sectional. Pregravid BMI (*p* = .045) positively predicted stress during the 2^nd^ trimester accounting for 2% of variance. Snack food intake (*p* = .002) significantly predicted stress during the 3^rd^ trimester, additionally accounting for 2% of variance.

Longitudinal. Pregnancy intention (*p* = .028) negatively predicted stress accounting for 3% of variance. Snack food intake of the 2^nd^ trimester did not emerge as a significant predictor of stress during the 3^rd^ trimester.

### Supplementary analyses

Supplementary hierarchical multiple linear regression analyses were calculated using the SES as an additional covariate in order to avoid data loss. It was found that the inclusion of SES in the 1^st^ block of the hierarchical regression analysis had no influence on the prediction of depressive symptoms by GWG, sleep problems, alcohol use, and cigarette smoking both cross-sectionally and longitudinally as well as on snack food intake cross-sectionally. Concerning the prediction of stress, the regression models including sleep problems, alcohol use, cigarette smoking, and snack food intake cross-sectionally and longitudinally as well as GWG and physical activity longitudinally, remained unchanged after including SES in the 1^st^ block. However, including SES as a covariate turned the previously non-significant 2^nd^ block to predict depressive symptoms (cross-sectionally and longitudinally) and stress (cross-sectionally) by physical activity significant and turned the previously significant predictors snack food intake and GWG to predict depressive symptoms and stress (cross-sectionally), respectively, insignificant (for detailed information, see Additional file 1: Table S1 and Additional file 2: Table S2).

Additionally, supplementary hierarchical multiple linear regression analyses were calculated using GWG as a covariate in the prediction of snack food intake by depressive symptoms and stress in order to consider significant positive correlation between snack food intake and depressive symptoms and stress cross-sectionally (all *p*s < .05). The results of the hierarchical multiple linear regression analyses to predict snack food intake by depressive symptoms and stress remained unchanged after controlling for GWG (all *p*s > .05).

## Discussion

To our knowledge, this is the first study that examined the associations between multiple maternal proximal risk factors, socio-demographic characteristics, pregnancy intention, and mental health problems in a large German population-based sample of pregnant women during their 2^nd^ and 3^rd^ trimester. While a number of proximal risk factors was related to mental health problems cross-sectionally, only sleep problems during the 2^nd^ trimester were associated with mental health problems in the 3^rd^ trimester, although this association was reduced when controlling for baseline depressive symptoms and stress. Consistent with previous findings [56–58], we found that women who planned pregnancy were less likely than those who did not plan pregnancy to show depressive symptoms and stress during the 2^nd^ and 3^rd^ trimester.

Contrary to our expectations, only snack food intake and sleep problems were significantly positively related to both depressive symptoms and stress cross-sectionally. Additionally, GWG was significantly positively linked to stress during the 2^nd^ trimester. However, GWG was not linked to stress after controlling for gestational age. The fact that women’s snack food intake was positively associated with women’s mental health problems in the 2^nd^ trimester of pregnancy is consistent with recent evidence in non-pregnant women and suggests that pregnancy does not have a specific effect on this association [20, 21]. Regarding sleep problems, the results are in line with previous research by Yu et al. demonstrating a cross-sectional association between sleep problems and elevated depressive symptoms in 1653 Chinese pregnant women assessed at the 1^st^, 2^nd^, and 3^rd^ trimester, with the strongest association found during the 2^nd^ trimester [59]. The result that GWG was positively related to stress in the 2^nd^ trimester extends previous evidence in pregnant women indicating that a GWG above the recommendations may not only be associated with higher distress in the postpartum [60] but already in the prenatal period. However, GWG was not related to depressive symptoms which might be related to the mild degree of depressive symptoms reported in our sample, potentially leading to an underestimation of the association.

Consistent with previous findings [61], sleep problems in the 2^nd^ trimester were significantly positively related to both depressive symptoms and stress in the 3^rd^ trimester. Because sleep is important to recover from stress, sleep problems may account for mental health problems during pregnancy [61]. Nevertheless, contrary to previous research indicating that sleep problems explain a small but significant additional variance in depressive symptoms after controlling for baseline depressive symptoms [61], sleep problems were not linked to mental health problems over and above depressive symptoms and stress assessed during the 2^nd^ trimester. The lack of explaining additional variance in mental health problems by sleep problems over and above baseline mental health problems scores in the current study may be due to differences between the assessments used to examine depressive symptoms (Beck Depression Inventory II [62] versus PHQ-D [42]). The fact that sleep problems are a criterion of depression may explain why, after controlling for mental health problems, sleep quality was not a significant predictor of both depressive symptoms and stress anymore. It is generally well known that there is a robust relationship between mental health and sleep problems and that similar underlying mechanisms explain this relationship [63]. The present finding emphasizes the relevance of assessing depressive symptoms and stress in the presence of sleep disorders during pregnancy and vice versa in order to avoid long-term adverse consequences for mother and child [63].

However, other proximal risk factors were not significantly associated with mental health problems in the cross-sectional and longitudinal analyses. First, the fact that physical activity did not emerge as a significant predictor in the current study might be related to the one-item self-report assessment of physical activity, which was a rather crude indicator of women’s actual physical activity because it is subject to item interpretation, socially desirable response behavior, and recall bias [64]. The zero result supports previous research, showing that the assessment of physical activity via self-report questionnaires versus rest and activity cycle monitoring mostly resulted in a failure to find a significant association between physical activity and mental health problems [30]. Furthermore, because the response categories do not reflect the intensity of physical activity, the results might be biased. Therefore, an objective assessment of physical activity, for example, by actigraphy, would be desirable to determine the actual time and intensity of physical activity. Second, for substance use, the low prevalence of cigarette smoking and alcohol use decreased the likelihood of detecting significant associations with maternal mental health problems with sufficient power. As substance use in pregnancy is a stigmatized behavior [65], the low prevalence may to some extent mirror socially desirable response behavior. Moreover, the low prevalence may additionally mirror the high socio-economic status of the current population [66].

In determining whether confounding variables were associated with mental health problems during pregnancy, we found pregravid BMI to be significantly positively related to depressive symptoms and stress during the 2^nd^ and 3^rd^ trimester. The result is in line with previous research in pregnant women showing that every unit increase in pregravid BMI was related to a 3% higher chance for experiencing perinatal depression [67]. While evidence in the general population suggests that higher BMI leads to stress resulting from stigmatization [68], financial, family [69], and health concerns [70], to our knowledge, the current study firstly evidenced an association between pregravid BMI and stress in pregnant women. Besides pregravid BMI, pregnancy intention was significantly negatively related to depressive symptoms during the 2^nd^ trimester and stress during the 2^nd^ and 3^rd^ trimester, in line with previous research indicating a 2- to 2.5-fold risk for depressive symptoms and stress in pregnant women during their 2^nd^ and 3^rd^ trimester who did not plan pregnancy [56–58]. Interestingly, pregnancy intention remained significantly negatively associated to stress over and above maternal depressive symptoms and stress assessed during the 2^nd^ trimester, indicating that an unplanned pregnancy causes remarkable mental burden. Consistently, Gipson, Koenig, and Hindin showed in their review that women who did not plan pregnancy tended to more strongly engage in health-impairing behaviors than women who planned pregnancy [71]. In addition, women with unintended pregnancies were, either directly or indirectly via health behaviors, at higher risk for adverse pregnancy outcomes [72]. In this context, practitioners should pay special attention to women with unintended pregnancies in order to prevent long-lasting complications for the mother and the child.

Supplementary analyses showed that a low SES combined with low physical activity predicted depressive symptoms and stress. It is well known that pregnant women with a lower SES reported higher levels of depressive symptoms [73] and stress [38], and were physically less active during pregnancy [74] than women at the higher end of the SES continuum. Furthermore, a low SES was found to turn the previously significant predictors snack food intake and GWG of depressive symptoms and stress insignificant, probably due to the already known association between low SES and increased GWG during pregnancy [75] and sweets consumption in women from the general population [76]. Overall, these results underlined the importance in pregnancy care to focus on pregnant women with low SES because they tend, either directly or indirectly through health-impairing behavior, to have higher levels of depressive symptoms and stress, which in turn encourage negative consequences for the health of the mother and the developing child in both the short- and long-term.

The prevalence of proximal risk factors like GWG and sleep problems in the present sample was largely comparable to that reported in previous pregnant samples [77–80]. The fact that more than half of the sample reported a daily snack food intake was consistent with previous findings revealing that 55% of pregnant women showed craving for sweets [81], one of the most common cravings during pregnancy [82]. However, the prevalence of substance use and insufficient physical activity in the present sample was slightly lower than previously described in the literature [83]. Concerning mental health problems, while we identified a slightly lower prevalence of major depression than previous studies using structured interviews and self-report questionnaires [3], the prevalence of experiencing at least one stressor was somewhat higher than previously self-reported in pregnant women [10], which might be a result of the high socio-economic status in our sample.

### Strengths, limitations, and future directions

A major strength of the present study is the large population-based sample assessed at two time points during pregnancy, via internationally established questionnaires with good psychometric properties to measure mental health problems [42, 48]. Other strengths include the absence of exclusion criteria to enhance generalizability and the objective measurement of weight and height during pregnancy. However, because pregnant women were not examined before pregnancy or during the 1^st^ trimester, controlling for a history of mental health problems in the cross-sectional analyses was not possible and generalizability of the results is limited to the 2^nd^ and 3^rd^ trimester of pregnancy. Further, the high socio-demographic status, typically observed in population-based studies of pregnant women [84, 85] could have attenuated the effects of proximal risk factors on maternal mental health problems. Although internationally established questionnaires were used to measure mental health problems, self-report measurement of mental health problems and proximal risk factors may underestimate actual health status [64, 86]. Objective measurement via standardized interviews and physiological/biochemical markers would have been desirable, but is not easy to implement in a population-based study.

This research is a foundation for future studies to further examine the mechanisms contributing to mental health problems during pregnancy. Based on the high prevalence of potentially adversely health-influencing risk factors during pregnancy, it is important to examine interventions that are best in minimizing these risk factors [87, 88]. Certainly, the results underline the importance to pay special attention to pregnant women with higher pregravid BMI, sleep problems, and especially to women who did not plan pregnancy as they are likely at risk for higher levels of depressive symptoms and stress. Because the variance in depressive symptoms and stress explained by maternal proximal risk factors was predominately found to be small, future research should identify other important variables, for example, low socio-economic status, social support, maternal anxiety, life stress, history of depression, and domestic violence [36]. Longitudinal studies are warranted to analyze patterns of proximal risk factors, depressive symptoms, and stress during the course of pregnancy and their short and long-lasting effects on the mother’s and child’s health.

## Conclusion

The results showed that pregravid BMI, sleep problems, and particularly pregnancy intention were related to maternal mental health problems during pregnancy. Future research should focus on designing longitudinal studies using standardized measures, particularly diagnostic interviews (e.g., Structured Clinical Interview for DSM-5 – Research Version [89]) and physiological or biochemical markers (e.g., cortisol [90, 91]), in order to analyze patterns of risk factors including depressive symptoms and stress during different stages of the perinatal period, and their short- and long-lasting effects on maternal and infantile health.

## Additional files

Additional file 1: **Table S1.** Results of hierarchical multiple linear regression analysis to predict depressive symptoms in the 2^nd^ and 3^rd^ trimester by proximal risk factors of the 2^nd^ trimester including the socio-economic status as a covariate. Results of supplementary hierarchical multiple linear regression analyses to predict depressive symptoms by including the socio-economic status as an additional covariate are displayed. (DOCX 26 kb)

Additional file 2: **Table S2.** Results of hierarchical multiple linear regression analysis to predict stress in the 2^nd^ and 3^rd^ trimester by proximal risk factors of the 2^nd^ trimester including the socio-economic status as a covariate. Results of supplementary hierarchical multiple linear regression analyses to predict stress by including the socio-economic status as an additional covariate are displayed. (DOCX 26 kb)

## Abbreviations

BMI: body mass index
GWG: gestational weight gain
LIFE: Leipzig Research Center for Civilization Diseases
PHQ: Patient Health Questionnaire
PSQI: Pittsburgh Sleep Quality Index
SES: socio-economic status
