# Supplementary material for: Gestational weight gain, physical activity, sleep problems, substance use, and food intake as proximal risk factors of stress and depressive symptoms during pregnancy
Source: BMC Pregnancy Childbirth. 2019 May 17;19:175. doi: 10.1186/s12884-019-2328-1 (PMC6525385; doi:10.1186/s12884-019-2328-1)
Supplement: Supplementary file 2 — Table S2. Results of hierarchical multiple linear regression analysis to predict stress in the 2nd and 3rd trimester by proximal risk factors of the 2nd trimester including the socio-economic status as a covariate. Results of supplementary hierarchical multiple linear regression analyses to predict stress by including the socio-economic status as an additional covariate are displayed. (DOCX 26 kb) [file 12884_2019_2328_MOESM2_ESM.docx]

**Additional file 2: Table S2. Results of hierarchical multiple linear regression analysis to predict stress in the 2^nd^ and 3^rd^ trimester by proximal risk factors of the 2^nd^ trimester including the socio-economic status as a covariate**

|  | *2^nd^ trimester – Cross-sectional* | | | | | |  | *3^rd^ trimester – Longitudinal* | | | | | |
| --- | --- | --- | --- | --- | --- | --- | --- | --- | --- | --- | --- | --- | --- |
| *Variable* | *n* | *B (SE)* | β | *R*^2^ | *F* | *R*^2^_change_ |  | *n* | *B (SE)* | β | *R*^2^ | *F* | *R*^2^_change_ |
| **GWG** | 302 |  |  |  |  |  |  | 231 |  |  |  |  |  |
| **Block 1** |  |  |  | 0.04 | 3.14* | 0.04 |  |  |  |  | 0.03 | 2.55 | 0.03 |
| Age |  | 0.03 (0.05) | 0.04 |  |  |  |  |  | – | – |  |  |  |
| Pregravid BMI |  | 0.05 (0.04) | 0.06 |  |  |  |  |  | 0.03 (0.05) | 0.04 |  |  |  |
| Pregnancy intention |  | –0.55 (0.46) | –0.07 |  |  |  |  |  | –0.95 (0.52) | –0.12 |  |  |  |
| SES |  | –0.13 (0.05) | –0.17* |  |  |  |  |  | –0.07 (0.05) | –0.09 |  |  |  |
| **Block 2** |  |  |  | 0.04 | 2.81* | 0.00 |  |  |  |  | 0.04 | 2.15 | 0.01 |
| GWG |  | 0.06 (0.05) | 0.07 |  |  |  |  |  | 0.05 (0.05) | 0.07 |  |  |  |
| **Physical activity** | 203 |  |  |  |  |  |  | 170 |  |  |  |  |  |

**Additional file 2: Table S2** **–** continued

|  | *2^nd^ trimester – Cross-sectional* | | | | | |  | *3^rd^ trimester – Longitudinal* | | | | | |
| --- | --- | --- | --- | --- | --- | --- | --- | --- | --- | --- | --- | --- | --- |
| *Variable* | *n* | *B (SE)* | β | *R*^2^ | *F* | *R*^2^_change_ |  | *n* | *B (SE)* | β | *R*^2^ | *F* | *R*^2^_change_ |
| **Block 1** |  |  |  | 0.03 | 1.48 | 0.03 |  |  |  |  | 0.01 | 0.65 | 0.01 |
| Age |  | 0.06 (0.05) | 0.08 |  |  |  |  |  | – | – |  |  |  |
| Pregravid BMI |  | 0.00 (0.05) | 0.00 |  |  |  |  |  | –0.02 (0.06) | –0.03 |  |  |  |
| Pregnancy intention |  | –0.54 (0.59) | –0.06 |  |  |  |  |  | –0.56 (0.64) | –0.07 |  |  |  |
| SES |  | –0.13 (0.06) | –0.16* |  |  |  |  |  | –0.05 (0.06) | –0.07 |  |  |  |
| **Block 2** |  |  |  | 0.07 | 2.21* | 0.04 |  |  |  |  | 0.05 | 1.41 | 0.04 |
| Physical activity |  |  |  |  | reference group: *once a week* | | | | |  |  |  |  |
| *Less than once a month* |  | 1.26 (0.52) | 0.18* |  |  |  |  |  | 1.07 (0.55) | 0.16 |  |  |  |

**Additional file 2: Table S2** **–** continued

|  | *2^nd^ trimester – Cross-sectional* | | | | | |  | *3^rd^ trimester – Longitudinal* | | | | | |
| --- | --- | --- | --- | --- | --- | --- | --- | --- | --- | --- | --- | --- | --- |
| *Variable* | *n* | *B (SE)* | β | *R*^2^ | *F* | *R*^2^_change_ |  | *n* | *B (SE)* | β | *R*^2^ | *F* | *R*^2^_change_ |
| *Once a month* |  | 0.06 (0.61) | 0.01 |  |  |  |  |  | 0.08 (0.65) | 0.01 |  |  |  |
| *Daily* |  | 1.55 (0.69) | 0.16* |  |  |  |  |  | 1.45 (0.77) | 0.15 |  |  |  |
| **Sleep problems** | 258 |  |  |  |  |  |  | 199 |  |  |  |  |  |
| **Block 1** |  |  |  | 0.05 | 3.38* | 0.05 |  |  |  |  | 0.05 | 3.17* | 0.05 |
| Age |  | 0.05 (0.05) | 0.07 |  |  |  |  |  | – | – |  |  |  |
| Pregravid BMI |  | 0.03 (0.05) | 0.04 |  |  |  |  |  | 0.01 (0.05) | 0.01 |  |  |  |
| Pregnancy intention |  | –0.80 (0.49) | –0.10 |  |  |  |  |  | –1.25 (0.54) | –0.17* |  |  |  |
| SES |  | –0.15 (0.05) | –0.19** |  |  |  |  |  | –0.08 (0.05) | –0.11 |  |  |  |
| **Block 2** |  |  |  | 0.11 | 6.49*** | 0.06 |  |  |  |  | 0.12 | 6.48*** | 0.07 |

**Additional file 2: Table S2** **–** continued

|  | *2^nd^ trimester – Cross-sectional* | | | | | |  | *3^rd^ trimester – Longitudinal* | | | | | |
| --- | --- | --- | --- | --- | --- | --- | --- | --- | --- | --- | --- | --- | --- |
| *Variable* | *n* | *B (SE)* | β | *R*^2^ | *F* | *R*^2^_change_ |  | *n* | *B (SE)* | β | *R*^2^ | *F* | *R*^2^_change_ |
| Sleep problems |  | 0.20 (0.05) | 0.26*** |  |  |  |  |  | 0.21 (0.05) | 0.28*** |  |  |  |
| **Alcohol use** | 267 |  |  |  |  |  |  | 209 |  |  |  |  |  |
| **Block 1** |  |  |  | 0.06 | 3.94** | 0.06 |  |  |  |  | 0.05 | 3.60* | 0.05 |
| Age |  | 0.03 (0.05) | 0.04 |  |  |  |  |  | – | – |  |  |  |
| Pregravid BMI |  | 0.06 (0.04) | 0.09 |  |  |  |  |  | 0.06 (0.05) | 0.08 |  |  |  |
| Pregnancy intention |  | –0.72 (0.47) | –0.09 |  |  |  |  |  | –1.12 (0.54) | –0.14* |  |  |  |
| SES |  | –0.14 (0.05) | –0.19** |  |  |  |  |  | –0.09 (0.05) | –0.11 |  |  |  |
| **Block 2** |  |  |  | 0.06 | 3.18** | 0.00 |  |  |  |  | 0.05 | 2.69* | 0.00 |

**Additional file 2: Table S2** **–** continued

|  | *2^nd^ trimester – Cross-sectional* | | | | | |  | *3^rd^ trimester – Longitudinal* | | | | | |
| --- | --- | --- | --- | --- | --- | --- | --- | --- | --- | --- | --- | --- | --- |
| *Variable* | *n* | *B (SE)* | β | *R*^2^ | *F* | *R*^2^_change_ |  | *n* | *B (SE)* | β | *R*^2^ | *F* | *R*^2^_change_ |
| Alcohol use |  |  |  |  | reference group: *non-drinker* | | | | |  |  |  |  |
| *Quitter* |  | –0.19 (0.42) | –0.03 |  |  |  |  |  | –0.04 (0.46) | –0.01 |  |  |  |
| **Cigarette smoking** | 238 |  |  |  |  |  |  | 185 |  |  |  |  |  |
| **Block 1** |  |  |  | 0.04 | 2.36 | 0.04 |  |  |  |  | 0.02 | 1.04 | 0.02 |
| Age |  | 0.07 (0.05) | 0.09 |  |  |  |  |  | – | – |  |  |  |
| Pregravid BMI |  | 0.05 (0.05) | 0.06 |  |  |  |  |  | 0.02 (0.05) | 0.02 |  |  |  |
| Pregnancy intention |  | –0.55 (0.55) | –0.06 |  |  |  |  |  | –0.76 (0.63) | –0.09 |  |  |  |
| SES |  | –0.14 (0.06) | –0.17* |  |  |  |  |  | –0.06 (0.06) | –0.08 |  |  |  |
| **Block 2** |  |  |  | 0.05 | 2.47* | 0.01 |  |  |  |  | 0.03 | 1.56 | 0.01 |

**Additional file 2: Table S2** **–** continued

|  | *2^nd^ trimester – Cross-sectional* | | | | | |  | *3^rd^ trimester – Longitudinal* | | | | | |
| --- | --- | --- | --- | --- | --- | --- | --- | --- | --- | --- | --- | --- | --- |
| *Variable* | *n* | *B (SE)* | β | *R*^2^ | *F* | *R*^2^_change_ |  | *n* | *B (SE)* | β | *R*^2^ | *F* | *R*^2^_change_ |
| Cigarette smoking |  |  |  |  | reference group: *non-smoker* | | | | |  |  |  |  |
| *Quitter* |  | 0.94 (0.56) | 0.11 |  |  |  |  |  | 1.11 (0.63) | 0.13 |  |  |  |
| **Snack food intake** | 253 |  |  |  |  |  |  | 195 |  |  |  |  |  |
| **Block 1** |  |  |  | 0.03 | 1.95 | 0.03 |  |  |  |  | 0.20 | 1.23 | 0.20 |
| Age |  | 0.04 (0.05) | 0.05 |  |  |  |  |  | – | – |  |  |  |
| Pregravid BMI |  | 0.05 (0.05) | 0.06 |  |  |  |  |  | 0.02 (0.05) | 0.03 |  |  |  |
| Pregnancy intention |  | –0.53 (0.50) | 0.05 |  |  |  |  |  | –0.79 (0.57) | –0.10 |  |  |  |
| SES |  | –0.10 (0.05) | –0.13 |  |  |  |  |  | –0.05 (0.06) | –0.07 |  |  |  |

**Additional file 2: Table S2** **–** continued

|  | *2^nd^ trimester – Cross-sectional* | | | | | |  | *3^rd^ trimester – Longitudinal* | | | | | |
| --- | --- | --- | --- | --- | --- | --- | --- | --- | --- | --- | --- | --- | --- |
| *Variable* | *n* | *B (SE)* | β | *R*^2^ | *F* | *R*^2^_change_ |  | *n* | *B (SE)* | β | *R*^2^ | *F* | *R*^2^_change_ |
| **Block 2** |  |  |  | 0.05 | 2.47* | 0.02 |  |  |  |  | 0.20 | 0.97 | 0.00 |
| Snack food intake |  | 0.33 (0.16) | 0.13* |  |  |  |  |  | 0.08 (0.18) | 0.03 |  |  |  |

*Note. n* number of pregnant women included in the model. *B (SE)* estimated value of raw (unstandardized) regression coefficient (standard error). β population value (standardized) of regression coefficient. *R^2^* coefficient of multiple determination. *F* Fisher’s ratio. BMI body mass index, kg/m^2^. SES socio-economic status of the family. GWG gestational weight gain. ^***^*p* < .001. ^**^*p* < .01. ^*^*p* < .05.
